# Supplementary material for: Burden of non-serious infections during biological use for rheumatoid arthritis
Source: PLoS One. 2024 Feb 20;19(2):e0296821. doi: 10.1371/journal.pone.0296821 (PMC10878515; doi:10.1371/journal.pone.0296821)
Supplement: S1 Appendix — (DOCX) [file pone.0296821.s001.docx]

**S1 Appendix**

**Burden of non-serious infections during biological use for rheumatoid arthritis**

Barbara Bergmans^1,2^, Naomi Jessurun^3^, Jette van Lint^3^, Jean-Luc Murk^2,4^, Eugène van Puijenbroek^3,5^, Esther de Vries^1,2^

1. Tranzo, Tilburg School of Social and Behavioral Sciences, Tilburg University, Tilburg, The Netherlands
2. Laboratory of Medical Microbiology and Immunology, Elisabeth-TweeSteden Hospital, Tilburg, The Netherlands
3. Netherlands Pharmacovigilance Centre Lareb, ’s-Hertogenbosch, The Netherlands
4. Microvida, Elisabeth-TweeSteden Hospital, Tilburg, The Netherlands
5. University of Groningen, Groningen Research Institute of Pharmacy, PharmacoTherapy, - Epidemiology & -Economics, Groningen, The Netherlands

Keywords: infection, biological, rheumatoid arthritis, secondary immunodeficiency, adverse drug reaction

**Index**

| **Appendix** | **Title** | **Page** |
| --- | --- | --- |
| Table 1 | MedDRA Preferred Terms and assigned organ systems (for infections considered to be possible, probable or definite by our team) | 3 |
| Table 2 | MedDRA Preferred Terms and assigned likely pathogen (for infections considered to be definite by our team) | 5 |
| Table 3 | Decisions made in database cleaning for inconsistencies in dates, weight, length, and age | 6 |
| Figure 1 | Percentage of patients still in follow-up per questionnaire | 7 |
| Table 4 | Characteristics of patients who completed only one questionnaire vs the remaining cohort | 8 |
| Figure 2 | Distribution of the time period between the start of the biological and the first study questionnaire | 9 |
| Figure 3 | Total number of questionnaires that were completed per calendar week number | 10 |
| Figure 4 | Affected organ systems in possible infections | 11 |
| Table 5 | Characteristics of possible infections in the cohort | 12 |
| Figure 5 | Impact scores per questionnaire for possible infections in various organ systems | 13 |
| Table 6 | All potential ADRs including infection label, divided across organ systems | 15 |
| Figure 6 | a. Number and b. proportion of probable and definite upper respiratory tract infections from the total number of reports | 17 |
| Figure 7 | Most likely pathogens in definite infections | 18 |
| Figure 8 | Upper respiratory tract infections subdivided into individual infection ADRs | 19 |
| Figure 9 | Definite and probable skin- and soft tissue infections subdivided into individual infection PTs and the proportion of contact with HCP per potential ADR | 20 |
| Table 7 | Contacts with healthcare professionals in definite and probable infections | 21 |
| Table 8 | Overview of possible, probable and definite infection PTs per biological | 22 |

**Table 1. MedDRA Preferred Terms and assigned organ systems (for infections considered to be possible, probable or definite by our team)**

| **Organ system** | **MedDRA Preferred Term** |
| --- | --- |
| Bone and joint | Joint swelling |
| Ear | Ear pain  Otitis media |
| Eye | Eye infection  Hordeolum  Blepharitis  Swelling of eyelid  Dacryocystitis |
| Gastro-intestinal tract | Gastroenteritis  Diverticulitis  Diarrhea  Enteritis  Vomiting |
| Genital tract | Vulvovaginal candidiasis  Anal candidiasis  Vulvitis |
| Lower respiratory tract | Pulmonary pain  Pneumonia  Lower respiratory tract infection fungal |
| Lymphatic tissue | Lymphadenopathy |
| Oral | Tongue fungal infection  Gingivitis  Periodontitis  Oral mucosal blistering  Oral pain  Oral fungal infection  Angular cheilitis  Glossodynia  Oral candidiasis  Stomatitis |
| Respiratory tract | Cough  Respiratory tract infection  Dyspnea  Productive cough  Lung disorder |
| Skin-soft tissue | Injection site erythema  Skin infection  Furuncle  Wound infection  Tinea faciei  Rash  Herpes zoster  Herpes dermatitis  Paronychia  Fungal skin infection  Anogenital warts  Erysipelas  Blister  Oral herpes  Acne  Injection site rash  Injection site induration  Cellulitis  Injection site inflammation  Staphylococcal infection  Bursitis  Skin papilloma  Injection site pain |
| Systemic | Body temperature increased  Infection susceptibility increased  Pyrexia  Influenza like illness  Malaise  Chills  Febrile neutropenia |
| Upper respiratory tract | Chronic sinusitis  Dysphonia  Nasal congestion  Nasal inflammation  Nasopharyngitis  Oropharyngeal pain  Pharyngitis  Rhinitis  Rhinorrhea  Sinus pain  Sinusitis  Sneezing  Throat irritation  Upper respiratory tract congestion  Upper respiratory tract infection  Upper respiratory tract inflammation |
| Urinary tract | Cystitis  Prostatic abscess  Pyelonephritis  Urinary tract discomfort |
| Other | Head discomfort |
| Unknown | Fungal infection  Inflammation  infection |

Patient-experienced adverse events were coded from System Organ Class (SOC) to Preferred Term (PT) using the Medical Dictionary for Regulatory Activities (MedDRA). As MedDRA SOCs do not necessarily convey information on the organ system in which a PT takes place, individual PTs were assigned an organ system by our team (BB, EdV, JLM and EvP).

PTs were then assigned a probability of being an infectious disease by our team. Discrepancies in rating decisions were resolved by discussion until consensus was reached. In the table above, only potential ADRs considered possible, probable or definite infections are included.

**Table 2. MedDRA Preferred Terms and assigned likely pathogen (for infections considered to be definite by our team)**

| **Likely pathogen** | **MedDRA Preferred Term** |
| --- | --- |
| Bacterial | Hordeolum  Cystitis  Furuncle  Sinusitis  Pneumonia  Wound infection  Prostatic abscess  Paronychia  Pyelonephritis  Erysipelas  Diverticulitis  Blepharitis  Periodontitis  Otitis media  Chronic sinusitis  Dacryocystitis  Staphylococcal infection |
| Viral | Herpes zoster  Herpes dermatitis  Anogenital warts  Oral herpes  Rhinitis  Skin papilloma |
| Fungal | Tinea faciei  Vulvovaginal candidiasis  Anal candidiasis  Tongue fungal infection  Fungal infection  Fungal skin infection  Oral fungal infection  Lower respiratory tract infection fungal  Oral candidiasis |
| Unknown | Eye infection  Skin infection  Pharyngitis  Upper respiratory tract infection  Respiratory tract infection  Nasopharyngitis |

Patient-experienced adverse events were coded from System Organ Class (SOC) to Preferred Term (PT) using the Medical Dictionary for Regulatory Activities (MedDRA). As MedDRA SOCs do not necessarily convey information on the causative micro-organism in infectious adverse events, individual PTs were assigned a most likely causative micro-organism by our team (BB, EdV, JLM and EvP). Discrepancies in rating decisions were resolved by discussion until consensus was reached.

Potential ADRs were then assigned a probability of being an infectious disease by our team. Discrepancies in rating decisions were resolved by discussion until consensus was reached. In the table above, only potential ADRs considered definite infections are included.

**Table 3. Decisions made in database cleaning for inconsistencies in dates, weight, length, and age**

| **Problem** | **No of incidents** | **Solution** |
| --- | --- | --- |
| Impossible numbers in weight/length/age category (for example, "weight 1840 kg") | 11 | If identified as an obvious typographical error, we changed the variable to the most likely alternative (6), otherwise the variable was excluded (5) |
| No weight/length/age filled out in Q1 | 3 | Exclusion of variable |
| Incorrectly written start dates of innovator (for example, "0000-01-00") or no start date of innovator filled in | 4 | Exclusion of variable |
| Impossible start dates of innovator (chronologically after biological start date) | 2 | Exclusion of variable |
| Incorrectly written start dates of ADR (for example, "0000-01-00") or no start date of potential ADRs | 1 | Exclusion of variable |
| Incorrectly written stop dates of potential ADR (for example, "0000-01-00") | 3 | Exclusion of variable |
| Impossible stop dates of potential ADRs (for example, in the future or in 1982) | 3 | If identified as an obvious typographical error, we changed the variable to the most likely alternative (2), otherwise the variable was excluded (1) |
| Start date of biological and start date of potential ADRs do not align (potential ADR chronologically before biological start date) | 12 | Inconsistent start dates, unclear which one is the culprit (1): exclusion of both variables  Start date of biological likely a mistake (2): exclusion of variable  Start date of biological in future (3): change variable to most likely alternative (1) or exclusion of variable (2)  Start date of ADR likely a mistake (2): exclusion of variable  Probable reversal of biological and potential ADR start dates (1): reversal of dates  Probable mistake in biological (unlikely switch) (1): exclusion of relevant questionnaire(s)  Patient admitted to having this potential ADR since before biological start (1): exclusion of potential ADR  Inconsistencies in multiple potential ADRs and biological start dates (1): exclusion of patient |
| Start date of potential ADR and stop date of potential ADR do not align (potential ADR stop date chronologically potential ADR start date) | 11 | Stop date potential ADR incorrect due to probable mistake in year/month (6): we changed the variable to the most likely alternative  Start date ADR incorrect due to probable mistake in year/month (2): we changed the variable to the most likely alternative  Inconsistent start date of potential ADR (1): exclusion of variable  Inconsistent stop date of potential ADR (1): exclusion of variable  Probable reversal of start and stop dates (1): reversal of start and stop dates |

**Figure 1. Percentage of patients still in follow-up per questionnaire**


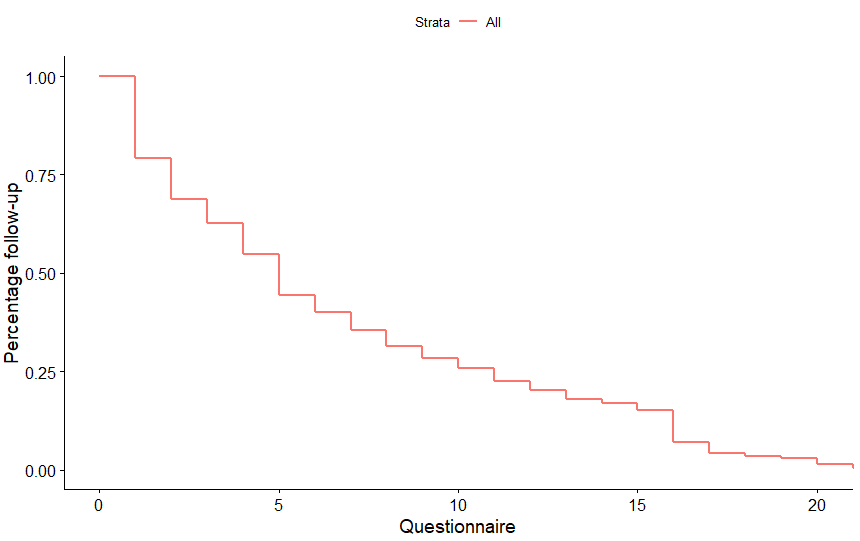


This figure shows the percentage of patients that filled in the corresponding number of questionnaires (on the x-axis). One fifth of participants (n=121, 20.6%) only completed one questionnaire, and more than half (n=326, 55.6%) stopped participating after the fifth questionnaire.

**Table 4. Characteristics of patients who completed only one questionnaire vs the remaining cohort**

|  | Completed only one questionnaire (mean, SD or n, %) | Completed more than one questionnaire (mean, SD or n, %) | p-value |
| --- | --- | --- | --- |
| Number of patients | 121 | 465 |  |
| Age | 58.0 (13.3) | 59.6 (11.8) | 0.15 |
| Weight | 78.2 (16.7) | 76.0 (14.4) | 0.19 |
| Length | 172 (10.0) | 172 (9.2) | 0.68 |
| Smoking status: never^a^ | 94 (77.7) | 396 (85.2) | 0.09 |
| Comorbidity present^b^ | 65 (53.7) | 288 (61.9) | 0.10 |
| Methotrexate | 58 (47.9) | 204 (43.9) | 0.42 |
| Azathioprine | 3 (2.5) | 14 (3.0) | 0.76 |
| Hydroxychloroquine | 11 (9.1) | 49 (10.5) | 0.64 |
| Hydrocortisone | 0 (0) | 9 (1.9) | 0.12 |
| Leflunomide | 6 (4.9) | 31 (6.7) | 0.49 |
| Prednisone | 15 (12.4) | 44 (9.5) | 0.34 |
| Prednisolone | 2 (1.7) | 21 (4.5) | 0.15 |
| Sulfasalazine | 7 (5.8) | 30 (6.5) | 0.79 |
| Methylprednisolone | 0 (0) | 6 (1.3) | 0.21 |
| No comedication | 29 (23.9) | 95 (20.4) | 0.40 |

Statistical analysis was performed by using either the Wilcoxon rank sum test for unpaired samples (age, weight, length) or the chi-square test.

^a^Compared were patients having never smoked versus patients that actively smoked or had smoked in the past or patients with an unknown smoking status

^b^Compared were patients having any comorbidity versus patients that had none or no information available

**Figure 2. Distribution of the time period between the start of the biological and the first study questionnaire**


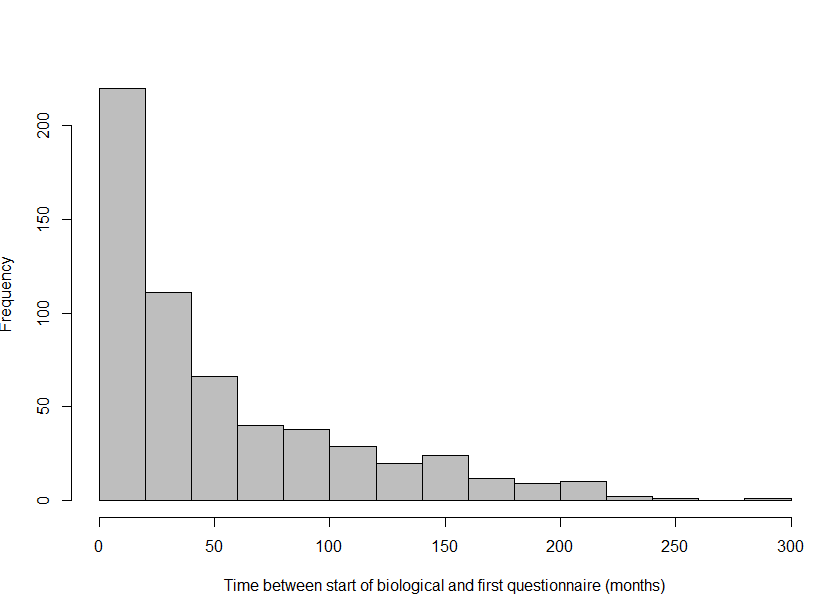


**Figure 3. Total number of questionnaires that were completed per calendar week number**


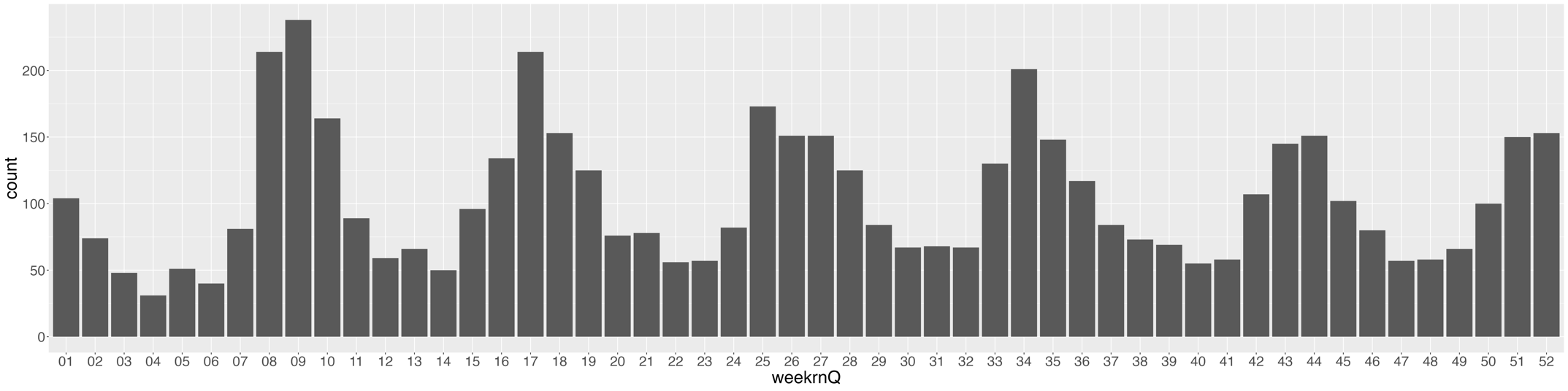


As can be seen in figure 3, questionnaires are often filled out in the same calendar week numbers. As patients were invited to fill out the questionnaires every two months, this results in a cyclical pattern.

**Figure 4. Affected organ systems in possible infections**


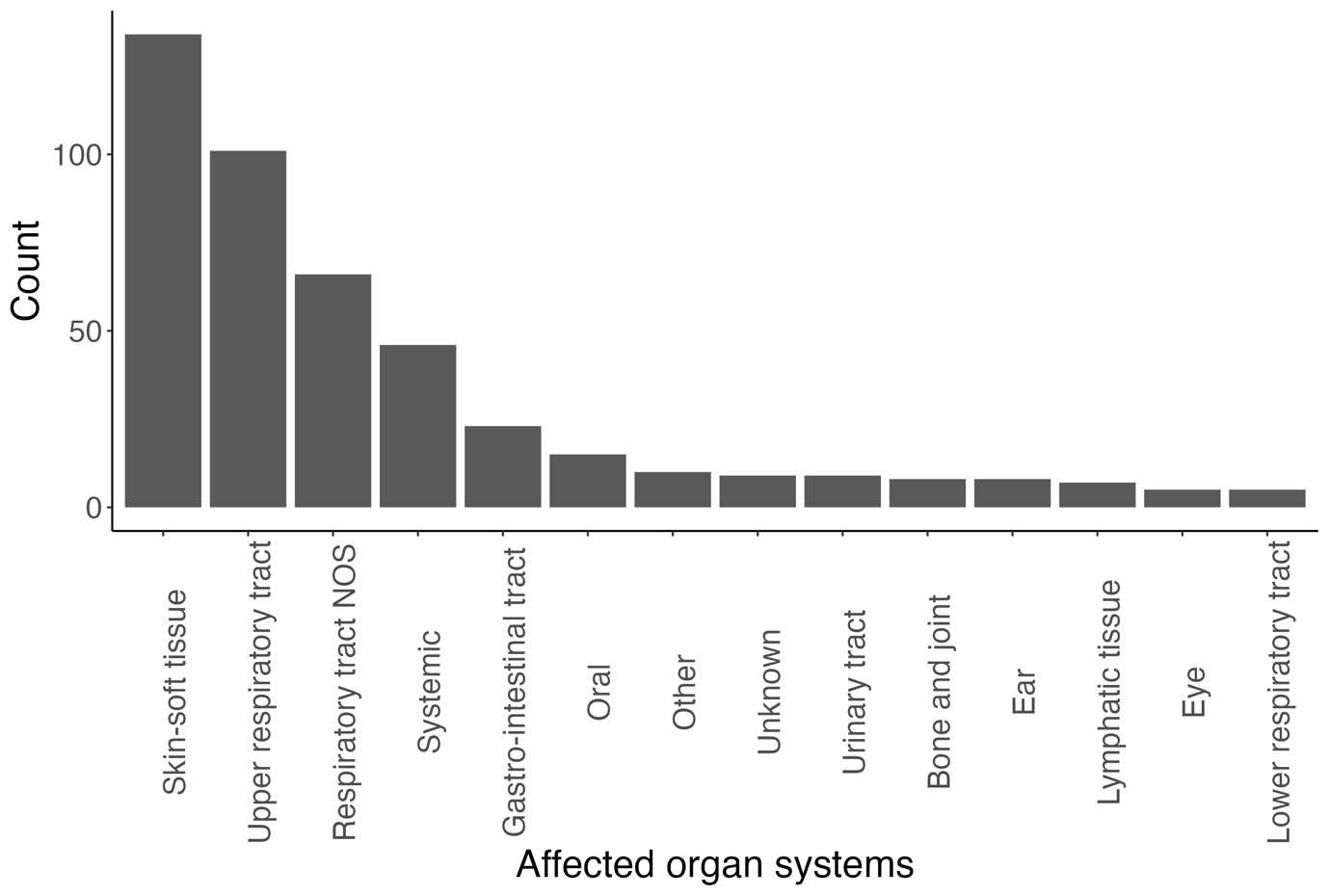


Only potential ADRs considered possible infections by our team are included in this figure.

**Table 5. Characteristics of possible infections in the cohort**

|  | Number of potential ADRs (n, %) | Number of occurrences per patient (median, IQR)^a^ | Impact score (median, IQR)^b^ | Time start biological – potential ADR (months) (median, IQR)^b,c^ | Duration (days)  (median, IQR)b | Contacted HCP (n, %) | Hospitalization (n, %) |
| --- | --- | --- | --- | --- | --- | --- | --- |
| **All infection-related potential ADRs^3^** | **867** | **3 (1.5-8.0)** | **2.0 (2.0-3.0)** | **16 (3.0-50.0)** | **32.5 (12.0-85.5)** | **350 (40.4)** | **12 (1.38)** |
| All possible infection-related potential ADRs^c^ | 446 (51.44) | 2.5 (2.0-5.0) | 2.0 (1.0-3.0) | 16.0 (3.0-45.0) | 45.0 (7.0-115.0) | 143 (32.1) | 4 (0.9) |

ADR= adverse drug reaction, HCP= Healthcare provider, IQR= interquartile range

^a^In a total of 156 patients reporting a possible, probable or infection

^b^The highest impact score, longest interval between start of the biological and potential ADR onset, and longest duration per potential ADR were chosen in unique ADRs per infection-patient.

^c^All infection-related potential ADRs: the total number of potential infection ADRs mentioned by all patients in all questionnaires (potential ADRs are counted each time when mentioned in a questionnaire by a patient)

**Figure 5. Impact scores per questionnaire for possible infections in various organ systems**


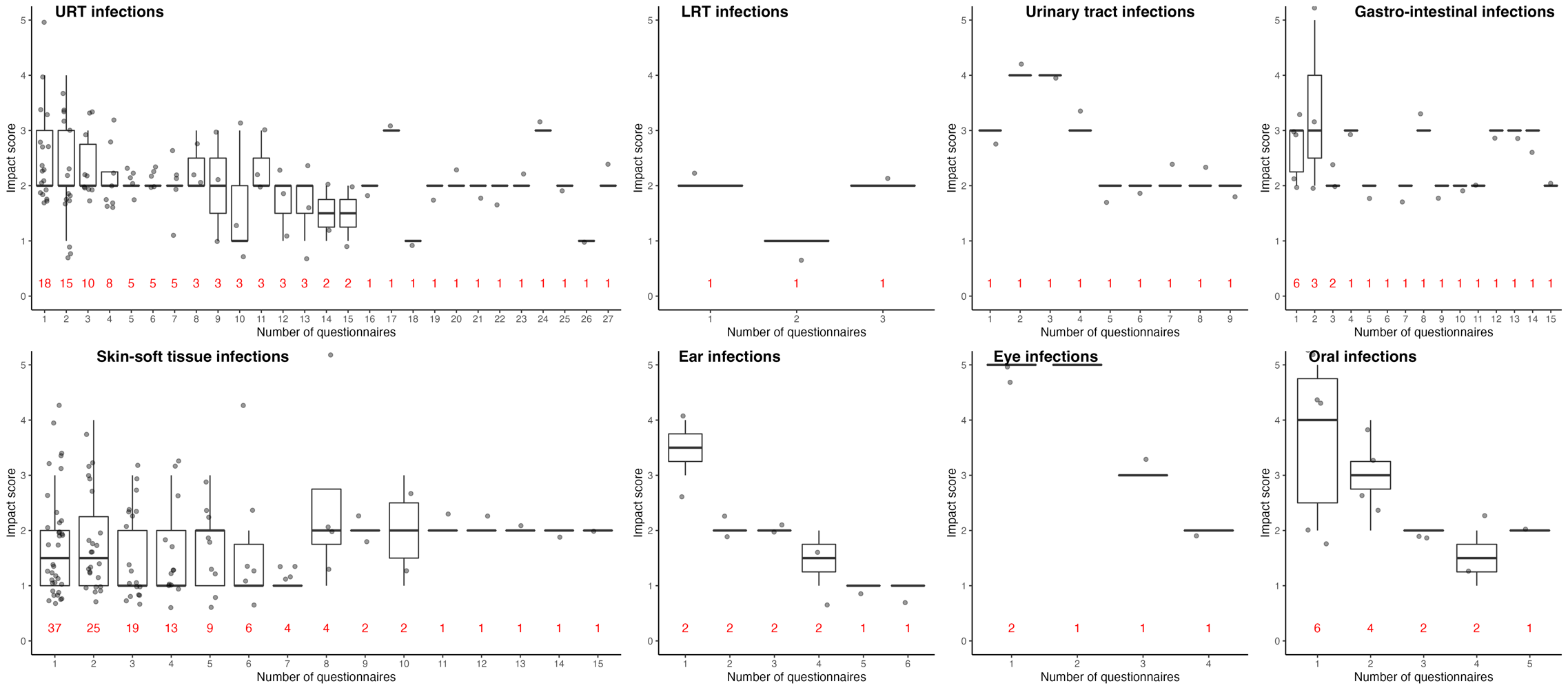


LRT= lower respiratory tract, URT= upper respiratory tract

Data are presented as patients reporting a probable or definite infection once or multiple times (x-axis) and the patient-assigned impact score (y-axis). The numbers in red represent the number of patients that reported an infection. The questionnaire in which a potential ADR was reported for the first time is indicated as “1” on the x-axis, the questionnaire in which a potential ADR in the same organ system was reported for the second time by that patient is indicated as “2” on the x-axis, etc. In some organ systems, e.g., URT infections, a single patient is observed that continues to mention a potential ADR in this organ system for an extended period of time.

**Table 6. All potential ADRs including infection label, divided across organ systems**

|  | Number of potential ADRs | Number of Patients^a^ | Time between start of biological and start of potential ADR (median, IQR) (months)^b^ | Potential ADR duration (median, IQR) (days)^b^ | Impact score (median, IQR) |
| --- | --- | --- | --- | --- | --- |
| **Respiratory tract infections** | **369** | **78** | **18.0 (4.0-56.0)** | **45.0 (15.0-151.0)** | **2.0 (2.0-3.0)** |
| Definite | 197 | 48 | 14.0 (5.0-63.0) | 33.5 (12.0-90.0) | 3.0 (2.0-3.0) |
| Probable | 0 | 0 | NA | NA | NA |
| Possible | 172 | 39 | 28.0 (4.0-56.0) | 80.0 (35.0-213.0) | 2.0 (2.0-3.0) |
| **Skin-soft tissue infections** | **213** | **58** | **9.5 (0.0-27.0)** | **31.0 (12.0-66.0)** | **2.0 (1.0-3.0)** |
| Definite | 75 | 23 | 24.0 (0.0-86.0) | 31.0 (14.0-61.0) | 3.0 (2.0-4.0) |
| Probable | 4 | 2 | 0.0 (0.0-88.0) | 27.0 (27.0-27.0) | 2.5 (1.5-3.5) |
| Possible | 134 | 37 | 3.0 (0.0-18.0) | 45.0 (2.0-120.0) | 1.0 (1.0-2.0) |
| **Systemic** | **88** | **31** | **6.0 (0.0-31.0)** | **22.5 (5.0 - 48.0)** | **3.0 (2.0-4.0)** |
| Definite | 0 | 0 | NA | NA | NA |
| Probable | 42 | 10 | 0.0 (0.0-6.0) | 43.0 (28.0-48.0) | 3.0 (2.0-3.0) |
| Possible | 46 | 22 | 14.0 (5.0-58.5) | 15.0 (5.0-45.0) | 3.0 (2.0-4.0) |
| **Urinary tract** | **36** | **16** | **12.0 (0.0-30.0)** | **12.0 (8.0 - 31.0)** | **3.0 (2.0 - 5.0)** |
| Definite | 27 | 15 | 22.0 (4.0-45.0) | 11.0 (7.5-26.0) | 3.0 (2.0-4.0) |
| Probable | 0 | 0 | NA | NA | NA |
| Possible | 9 | 1 | 11.0 (11.0-11.0) | 90.0 (90.0-90.0) | 2.0 (2.0-3.0) |
| **Oral** | **30** | **9** | **25.0 (15.0-42.0)** | **49.0 (23.0-92.0)** | **2.0 (2.0-4.0)** |
| Definite | 15 | 4 | 42.0 (2.0-42.0) | 49.0 (23.0-78.0) | 2.0 (2.0-3.0) |
| Probable | 0 | 0 | NA | NA | NA |
| Possible | 15 | 6 | 15.0 (15.0-25.0) | 61.50 (24.0-99.0) | 2.0 (2.0-4.0) ( |
| **Genital tract** | **29** | **5** | **80.0 (4.0 - 80.0)** | **64.0 (52.0 - 245.0)** | **3.0 (2.0-3.0)** |
| Definite | 14 | 4 | 2.0 (0.0-98.0) | 64.0 (52.0-245.0) | 3.0 (2.0-4.0) |
| Probable | 15 | 1 | 80.0 (80.0-80.0) | NA | 3.0 (3.0-3.0) |
| Possible | 0 | 0 | NA | NA | NA |
| **Gastro-intestinal tract** | **26** | **8** | **50.0 (33.0-50.0)** | **8.0 (0.0-91.00)** | **3.0 (2.0-3.0)** |
| Definite | 1 | 1 | 23.0 (23.0-23.0) | NA | NA |
| Probable | 2 | 1 | 33.0 (33.0-33.0) | 15.0 (15.0-15.0) | 4.0 (4.0-4.0) |
| Possible | 23 | 6 | 50.0 (35.0-50.0) | 1.0 (1.0-91.0) | 2.5 (2.0-3.0) |
| **Eye** | **24** | **11** | **48.0 (11.0-79.0)** | **23.0 (13.0-49.0)** | **3.0 (2.0-4.0)** |
| Definite | 19 | 10 | 52.0 (29.0-79.0) | 25.5 (13.0-49.0) | 3.0 (2.0-3.0) |
| Probable | 0 | 0 | NA | NA | NA |
| Possible | 5 | 2 | 11.0 (11.0-11.0) | 14.0 (14.0-14.0) | 5.0 (3.0-5.0) |
| **Ear** | **11** | **3** | **18.0 (18.0-56.0)** | **105.0 (99.0-111.0)** | **2.0 (1.0-3.0)** |
| Definite | 3 | 1 | 56.0 (56.0-56.0) | 99.0 (99.0-99.0) | 2.0 (1.0-5.0) |
| Probable | 0 | 0 | NA | NA | NA |
| Possible | 8 | 2 | 18.0 (18.0-18.0) | 111.0 (111.0-111.0) | 2.0 (1.5-2.5) |
| **Bone and joint** | **8** | **1** | **19.0 (19.0-19.0)** | **NA** | **2.0 (1.5-2.5)** |
| Definite | 0 | 0 | NA | NA | NA |
| Probable | 0 | 0 | NA | NA | NA |
| Possible | 8 | 1 | 19.0 (19.0-19.0) | NA | 2.0 (1.5-2.5) |
| **Lymphatic tissue** | **7** | **1** | **1.0 (1.0-1.0)** | **NA** | **2.0 (1.0-2.0)** |
| Definite | 0 | 0 | NA | NA | NA |
| Probable | 0 | 0 | NA | NA | NA |
| Possible | 7 | 1 | 1.0 (1.0-1.0) | NA | 2.0 (1.0-2.0) |
| **Other** | **10** | **1** | **50.0 (18.0-50.0)** | **879.0 (879.0-879.0)** | **3.0 (2.0-3.0)** |
| Definite | 0 | 0 | NA | NA | NA |
| Probable | 0 | 0 | NA | NA | NA |
| Possible | 10 | 1 | 37.20 (16.52) | 879 (0) | 2.70 (0.95) |
| **Unknown** | **17** | **5** | **38.0 (0.0-153.0)** | **177.0 (177.0 - 177.0)** | **2.0 (2.0-3.0)** |
| Definite | 8 | 2 | 0.0 (0.0-38.0) | NA | 2.5 (2.0-3.0) |
| Probable | 0 | 0 | NA | NA | NA |
| Possible | 9 | 3 | 50.0 (18.0-50.0) | 879.0 (879.0-879.0) | 3.0 (1.0-3.0) |

ADR= Adverse Drug Reaction, IQR= interquartile range, PT= MedDRA Preferred Term;

^a^Number of distinct patients reporting a potential infection PT in a specific organ system. A patient can report multiple infections in the same organ system that have different labels (possible, probable or definite infection), therefore, the numbers of patients within one organ system do not necessarily add up.

^b^Timespan between biological start and start of the potential ADR and potential ADR duration were calculated using patient-provided start and stop dates, if available.

This table shows all potential ADRs in the dataset, and in how many patients they occurred. Potential ADRs are divided across the organ systems to which they were assigned by our team, and further subdivided by the infection label they were assigned by our team (possible, probable or definite infection). Per row, information on the timespan between the start of the biological and the start of the potential ADR, the duration of the potential ADR and its median impact score is shown.

**Figure 6. a. Number and b. proportion of probable and definite upper respiratory tract infections from the total number of reports**

**
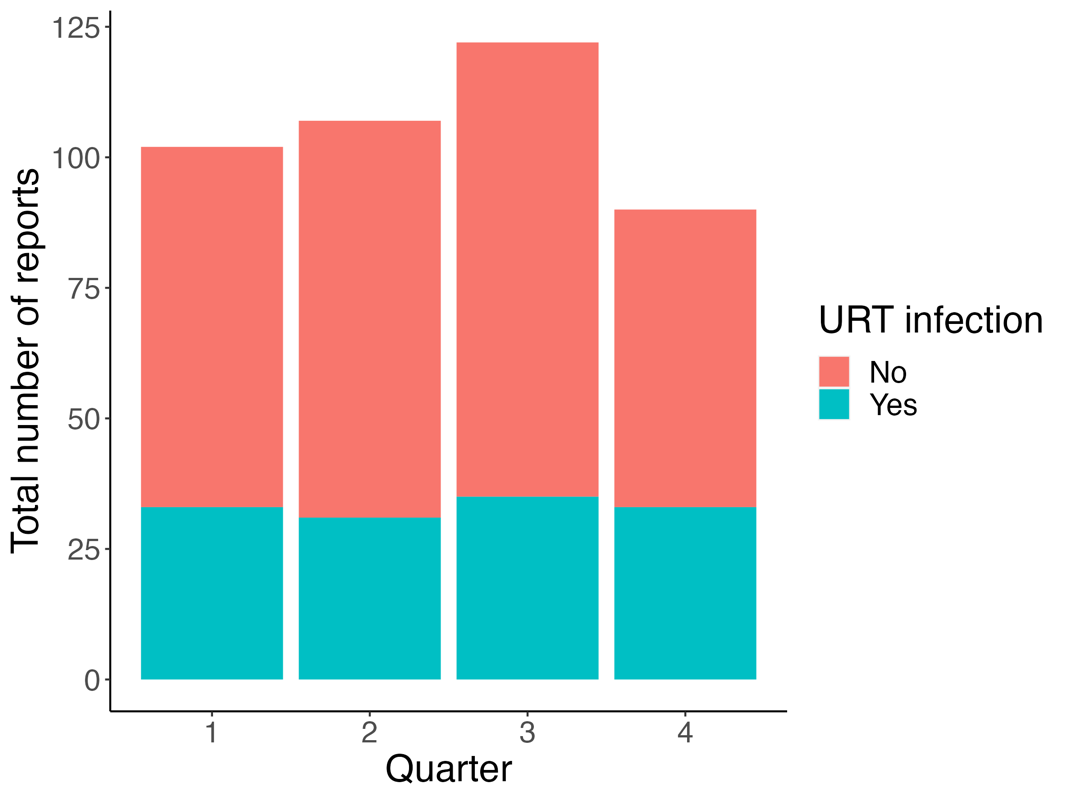

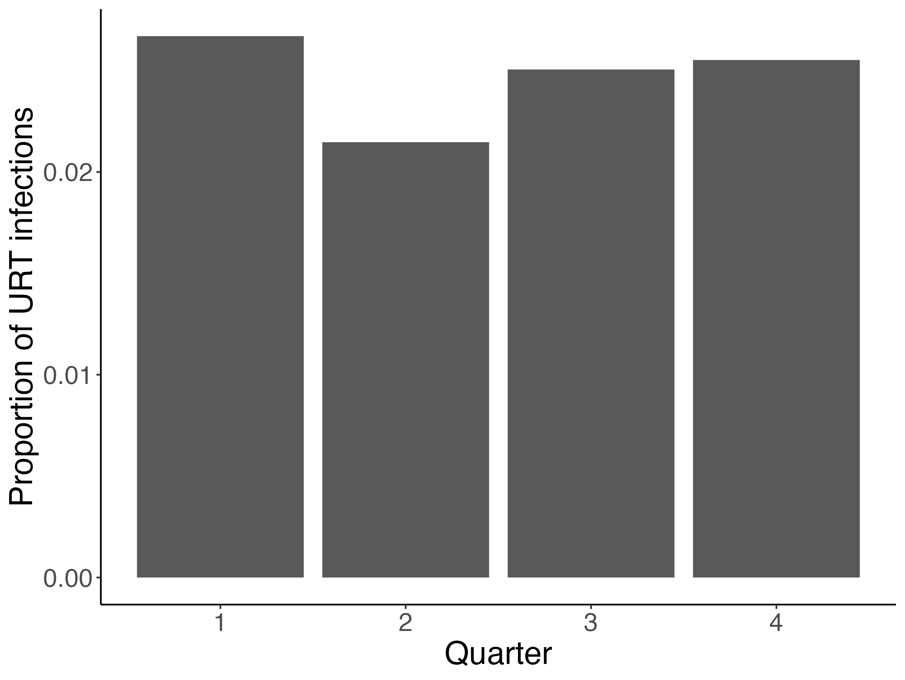
**

URT = upper respiratory tract

An analysis of probable and definite upper respiratory tract infections in different year-quarters.

Questionnaire fill-in dates had a clear cyclical pattern (see appendix S7), therefore we chose to visualize the proportion of upper respiratory tract infections per year-quarter. The total number of reports signifies all reports, including potential non-infectious ADRs and reports where patients described no potential ADRs.

**Figure 7. Most likely pathogens in definite infections**


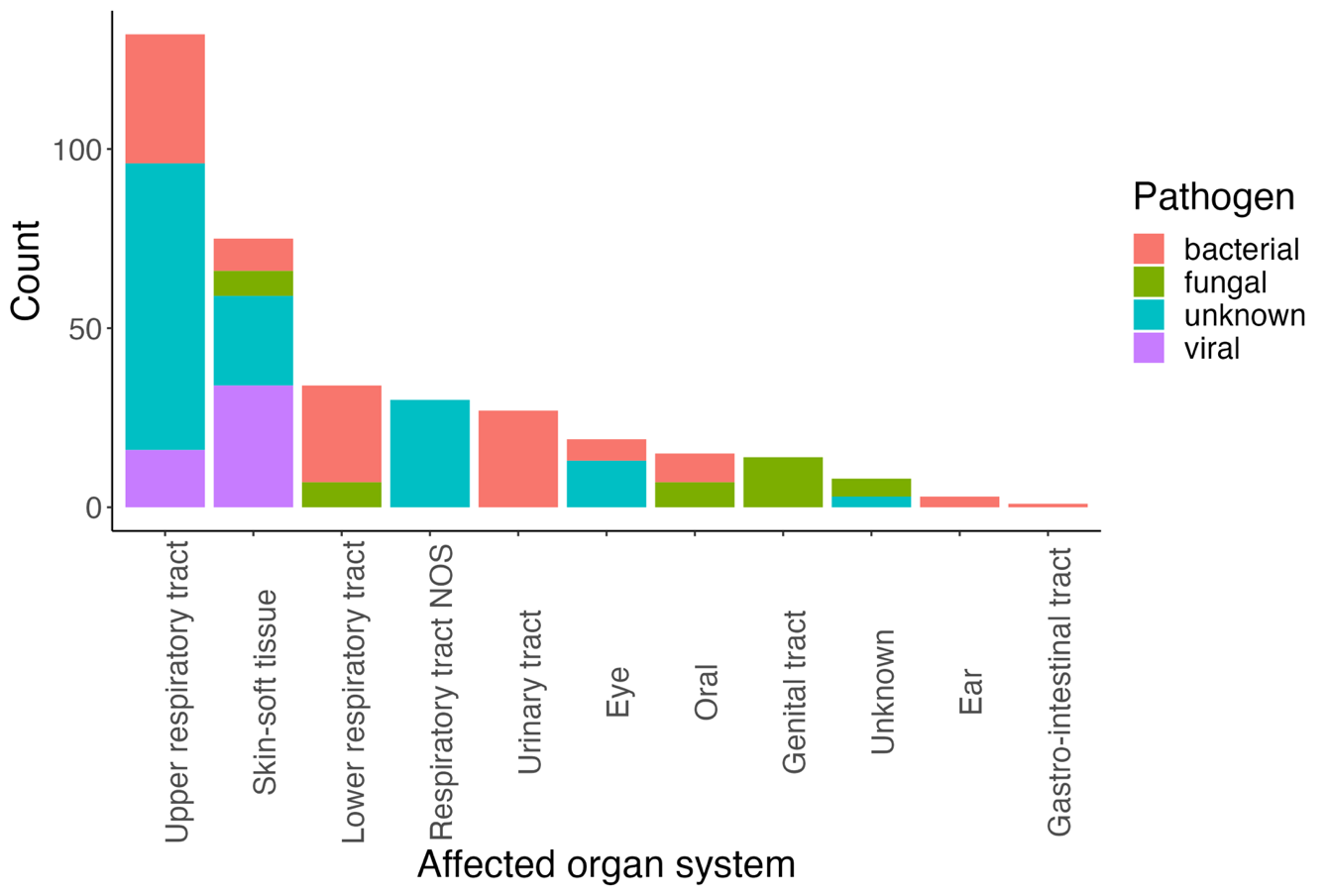


Only potential ADRs considered definite infections by our team were included in this figure.

**Figure 8. Upper respiratory tract infections subdivided into individual potential infectious ADRs**


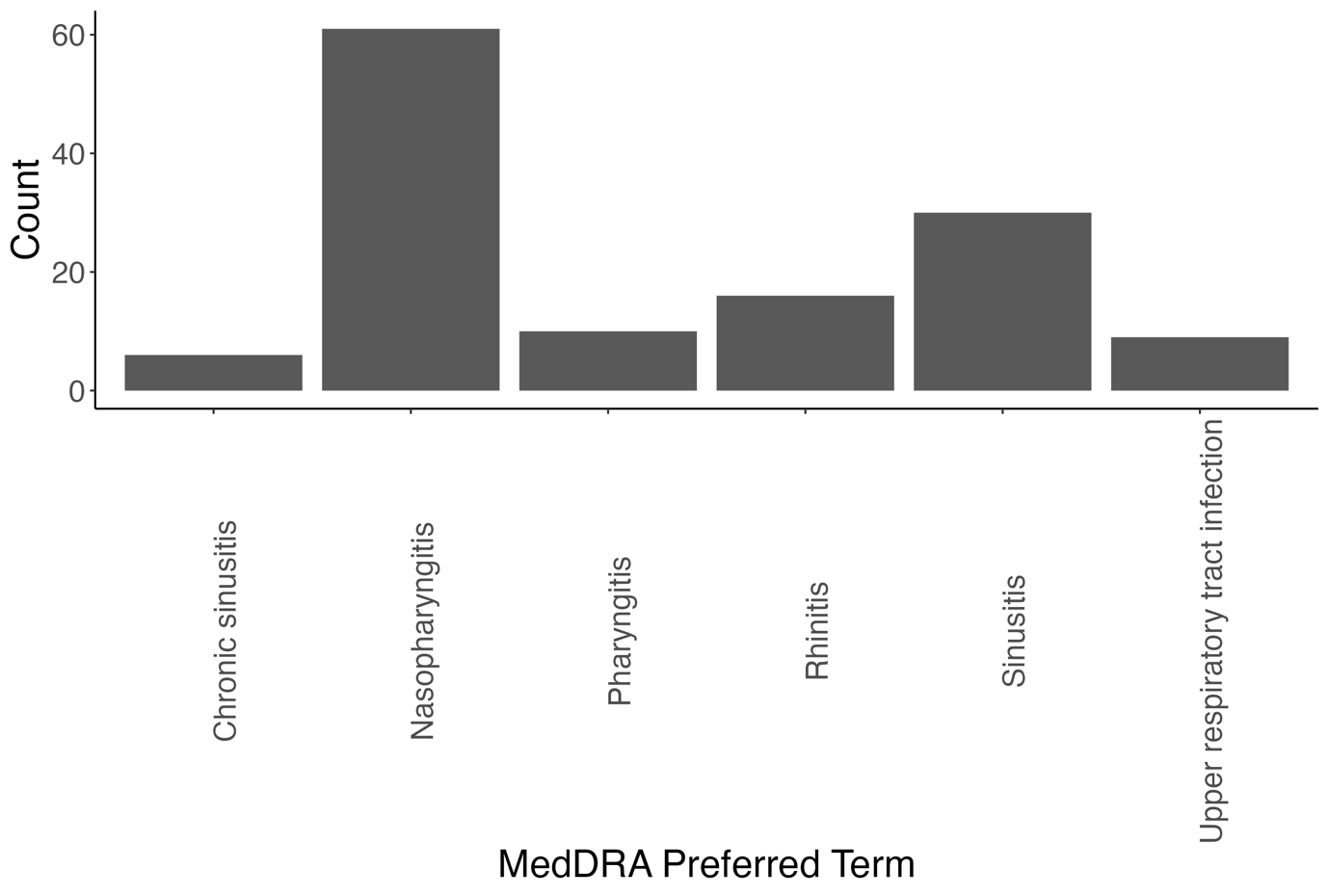


This figure shows the subdivision of individual potential infectious ADRs in the upper respiratory tract that were considered probable or definite infections by our team.

**Figure 9. Definite and probable skin- and soft tissue infections subdivided into individual infection PTs and the proportion of contact with HCP per potential ADR**

**
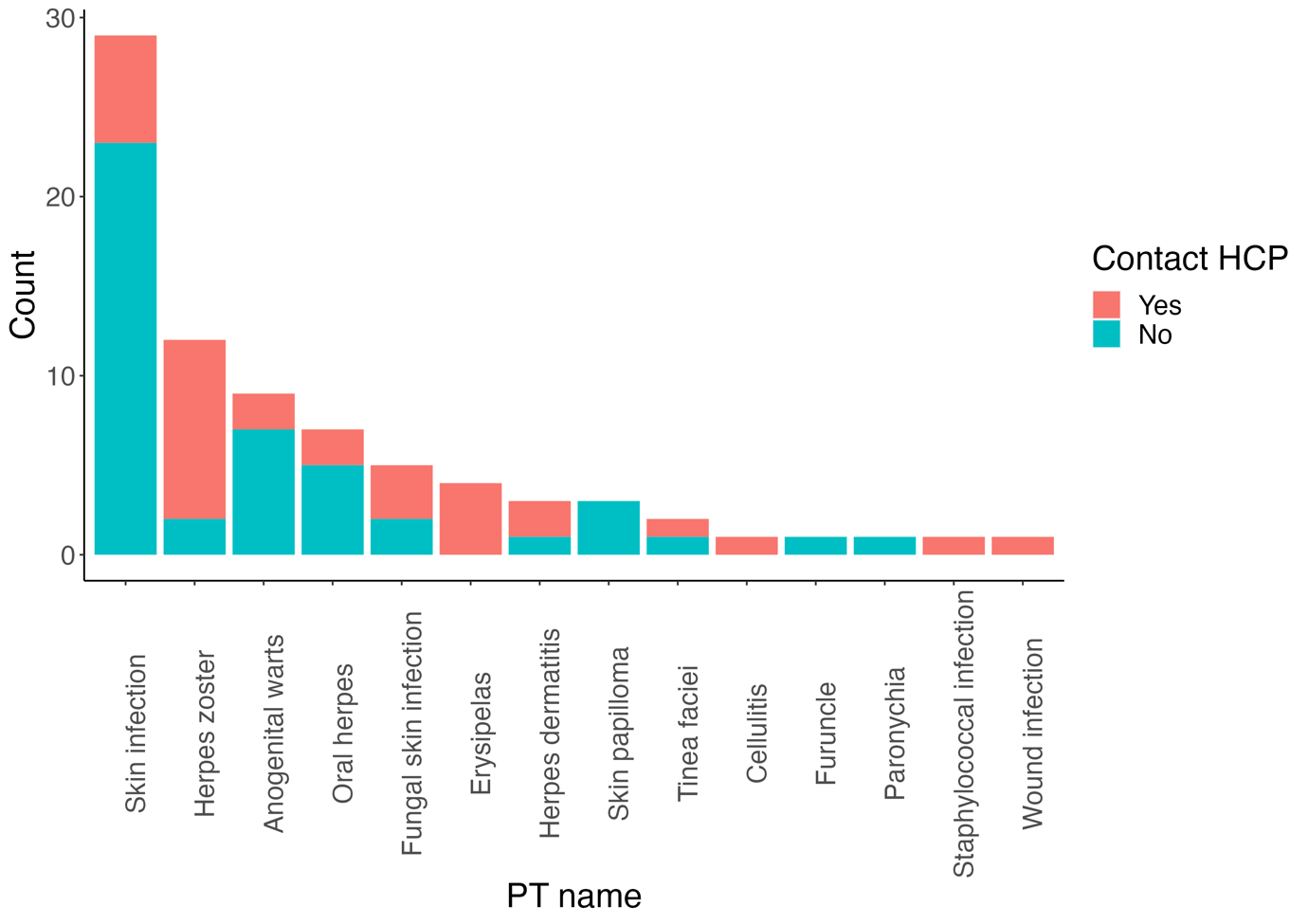
**

ADR = adverse drug reaction, HCP = healthcare professional, PT = MedDRA preferred term

This figure shows the subdivision of individual infection PTs in the skin and soft tissues that were considered probable or definite infections by our team, and the proportion of contact with an HCP per PT. Skin infection (not otherwise specified) occurs most frequently at a total of 29 events and has a low rate of HCP contact.

**Table 7. Contacts with healthcare professionals in definite and probable infections**

| **Organ system** | **Contact HCP (n, %)** | **HCP specific (n, %)^a^** |
| --- | --- | --- |
| Upper respiratory tract | 43 (32.6) | GP 22 |
|  |  | Medical specialist 24 |
|  |  | Nurse 8 |
| Lower respiratory tract | 27 (79.4) | GP 15 |
|  |  | Medical specialist 18 |
|  |  | Nurse 3 |
|  |  | Pharmacist 5 |
|  |  | Other 1 |
| Skin-soft tissue | 33 (41.8) | GP 20 |
|  |  | Medical specialist 20 |
|  |  | Nurse 6 |
|  |  | Pharmacist 1 |
| Systemic | 22 (52.4) | GP 21 |
|  |  | Medical specialist 11 |
|  |  | Nurse 5 |
|  |  | Pharmacist 1 |
| Urinary tract | 19 (70.4) | GP 17 |
|  |  | Medical specialist 7 |
|  |  | Nurse 3 |
|  |  | Pharmacist 2 |
| Oral | 7 (46.7) | GP 4 |
|  |  | Medical specialist 2 |
|  |  | Nurse 1 |
|  |  | Other: dentist 3 |
| Genital tract | 16 (55.17) | GP 9 |
|  |  | Medical specialist 8 |
|  |  | Pharmacist 1 |
| Gastro-intestinal tract | 2 (66.7) | GP 1 |
|  |  | Medical specialist 1 |
|  |  | Nurse 1 |
|  |  | Other 1 |
| Eye | 13 (72.2) | GP 11 |
|  |  | Medical specialist 3 |
|  |  | Other 1 |
| Ear | 3 (100) | GP 1 |
|  |  | Medical specialist 3 |
| Unknown | 3 (37.5) | GP 1 |
|  |  | Medical specialist 2 |
|  |  | Other 1 |

GP= general practitioner, HCP= healthcare provider,

^a^Patients could contact multiple HCPs.

This table shows the percentage of PTs for which contact with a HCP was sought, and the specific HCPs that were contacted per organ system.

**Table 8. Overview of possible, probable and definite infection PTs per biological**

|  | **Abatacept** | **Adalimumab** | **Anakinra** | **Certolizumab pegol** | **Etanercept** | **Golimumab** | **Infliximab** | **Rituximab** | **Sarilumab** | **Secukinumab** | **Tocilizumab** | **Vedolizumab** |
| --- | --- | --- | --- | --- | --- | --- | --- | --- | --- | --- | --- | --- |
| **Acne** | 0 | 0 | 0 | 0 | 3 | 0 | 0 | 0 | 0 | 0 | 0 | 0 |
| **Anal candidiasis** | 0 | 0 | 0 | 0 | 3 | 0 | 0 | 1 | 0 | 0 | 0 | 0 |
| **Angular cheilitis** | 0 | 0 | 0 | 0 | 0 | 0 | 0 | 0 | 0 | 0 | 4 | 0 |
| **Anogenital warts** | 0 | 0 | 0 | 4 | 5 | 0 | 0 | 0 | 0 | 0 | 0 | 0 |
| **Blepharitis** | 0 | 0 | 0 | 0 | 1 | 0 | 0 | 0 | 0 | 0 | 0 | 0 |
| **Blister** | 0 | 0 | 0 | 1 | 0 | 0 | 0 | 4 | 0 | 0 | 0 | 0 |
| **Body temperature increased** | 0 | 2 | 0 | 0 | 0 | 0 | 0 | 0 | 0 | 0 | 0 | 0 |
| **Bursitis** | 0 | 0 | 0 | 0 | 0 | 0 | 0 | 1 | 0 | 0 | 0 | 0 |
| **Cellulitis** | 0 | 0 | 0 | 0 | 0 | 0 | 0 | 1 | 0 | 0 | 0 | 0 |
| **Chills** | 0 | 2 | 0 | 0 | 4 | 0 | 0 | 0 | 0 | 0 | 0 | 0 |
| **Chronic sinusitis** | 0 | 0 | 0 | 0 | 6 | 0 | 0 | 0 | 0 | 0 | 0 | 0 |
| **Cough** | 2 | 17 | 0 | 1 | 10 | 0 | 0 | 3 | 0 | 0 | 3 | 0 |
| **Cystitis** | 0 | 9 | 0 | 3 | 8 | 1 | 0 | 0 | 0 | 0 | 1 | 0 |
| **Dacryocystitis** | 0 | 0 | 0 | 0 | 1 | 0 | 0 | 0 | 0 | 0 | 0 | 0 |
| **Diarrhoea** | 0 | 2 | 0 | 0 | 19 | 0 | 0 | 0 | 0 | 0 | 0 | 0 |
| **Diverticulitis** | 0 | 1 | 0 | 0 | 0 | 0 | 0 | 0 | 0 | 0 | 0 | 0 |
| **Dysphonia** | 0 | 0 | 0 | 13 | 0 | 0 | 0 | 0 | 0 | 0 | 0 | 0 |
| **Dyspnoea** | 0 | 0 | 0 | 0 | 11 | 0 | 0 | 0 | 0 | 0 | 0 | 0 |
| **Ear pain** | 0 | 0 | 0 | 0 | 8 | 0 | 0 | 0 | 0 | 0 | 0 | 0 |
| **Enteritis** | 0 | 0 | 0 | 0 | 1 | 0 | 0 | 0 | 0 | 0 | 0 | 0 |
| **Erysipelas** | 0 | 0 | 0 | 1 | 1 | 0 | 0 | 1 | 0 | 0 | 1 | 0 |
| **Eye infection** | 0 | 0 | 0 | 0 | 8 | 0 | 0 | 5 | 0 | 0 | 0 | 0 |
| **Eye irritation** | 0 | 0 | 0 | 0 | 4 | 0 | 0 | 0 | 0 | 0 | 0 | 0 |
| **Febrile neutropenia** | 0 | 0 | 0 | 1 | 0 | 0 | 0 | 0 | 0 | 0 | 0 | 0 |
| **Fungal infection** | 0 | 0 | 0 | 5 | 0 | 0 | 0 | 0 | 0 | 0 | 0 | 0 |
| **Fungal skin infection** | 0 | 5 | 0 | 0 | 0 | 0 | 0 | 0 | 0 | 0 | 0 | 0 |
| **Furuncle** | 0 | 1 | 0 | 0 | 0 | 0 | 0 | 0 | 0 | 0 | 0 | 0 |
| **Gastroenteritis** | 0 | 0 | 0 | 2 | 0 | 0 | 0 | 0 | 0 | 0 | 0 | 0 |
| **Gingivitis** | 0 | 0 | 0 | 5 | 0 | 0 | 0 | 0 | 0 | 0 | 0 | 0 |
| **Glossodynia** | 0 | 0 | 0 | 0 | 0 | 1 | 0 | 0 | 0 | 0 | 0 | 0 |
| **Head discomfort** | 0 | 0 | 0 | 0 | 10 | 0 | 0 | 0 | 0 | 0 | 0 | 0 |
| **Herpes dermatitis** | 0 | 0 | 0 | 1 | 2 | 0 | 0 | 0 | 0 | 0 | 0 | 0 |
| **Herpes zoster** | 0 | 1 | 0 | 0 | 6 | 0 | 0 | 2 | 0 | 0 | 3 | 0 |
| **Hordeolum** | 0 | 4 | 0 | 0 | 0 | 0 | 0 | 0 | 0 | 0 | 0 | 0 |
| **Infection** | 0 | 0 | 0 | 0 | 3 | 0 | 0 | 0 | 0 | 0 | 0 | 0 |
| **Infection susceptibility increased** | 0 | 25 | 0 | 0 | 10 | 0 | 0 | 0 | 0 | 0 | 5 | 1 |
| **Inflammation** | 0 | 6 | 0 | 0 | 2 | 1 | 0 | 0 | 0 | 0 | 0 | 0 |
| **Influenza like illness** | 0 | 4 | 0 | 1 | 15 | 0 | 0 | 0 | 0 | 0 | 0 | 0 |
| **Injection site erythema** | 3 | 14 | 0 | 1 | 47 | 5 | 0 | 0 | 0 | 0 | 5 | 0 |
| **Injection site induration** | 0 | 0 | 0 | 0 | 2 | 0 | 0 | 0 | 0 | 0 | 0 | 0 |
| **Injection site inflammation** | 0 | 2 | 0 | 0 | 10 | 0 | 0 | 0 | 0 | 0 | 0 | 0 |
| **Injection site pain** | 0 | 0 | 0 | 0 | 10 | 0 | 0 | 0 | 0 | 0 | 0 | 0 |
| **Injection site rash** | 0 | 0 | 0 | 0 | 2 | 0 | 0 | 0 | 0 | 0 | 0 | 0 |
| **Joint swelling** | 0 | 0 | 0 | 0 | 8 | 0 | 0 | 0 | 0 | 0 | 0 | 0 |
| **Lower respiratory tract infection fungal** | 0 | 0 | 0 | 0 | 7 | 0 | 0 | 0 | 0 | 0 | 0 | 0 |
| **Lung disorder** | 0 | 0 | 0 | 0 | 15 | 0 | 0 | 0 | 0 | 0 | 0 | 0 |
| **Lymphadenopathy** | 0 | 0 | 0 | 0 | 0 | 0 | 0 | 7 | 0 | 0 | 0 | 0 |
| **Malaise** | 1 | 0 | 0 | 0 | 4 | 0 | 0 | 0 | 0 | 0 | 0 | 0 |
| **Nasal congestion** | 0 | 18 | 0 | 0 | 0 | 0 | 0 | 0 | 0 | 0 | 0 | 0 |
| **Nasal inflammation** | 0 | 0 | 0 | 0 | 0 | 0 | 0 | 0 | 0 | 0 | 1 | 0 |
| **Nasopharyngitis** | 1 | 22 | 0 | 1 | 26 | 0 | 0 | 7 | 0 | 0 | 4 | 0 |
| **Oral candidiasis** | 0 | 0 | 0 | 0 | 0 | 0 | 0 | 0 | 2 | 0 | 0 | 0 |
| **Oral fungal infection** | 0 | 0 | 0 | 0 | 3 | 0 | 0 | 0 | 0 | 0 | 0 | 0 |
| **Oral herpes** | 0 | 0 | 0 | 0 | 7 | 0 | 0 | 0 | 0 | 0 | 0 | 0 |
| **Oral mucosal blistering** | 0 | 0 | 0 | 0 | 0 | 0 | 0 | 0 | 0 | 0 | 2 | 0 |
| **Oral pain** | 0 | 0 | 0 | 0 | 0 | 0 | 0 | 0 | 1 | 0 | 1 | 0 |
| **Oropharyngeal pain** | 0 | 2 | 0 | 0 | 1 | 0 | 0 | 0 | 1 | 0 | 3 | 0 |
| **Otitis media** | 0 | 3 | 0 | 0 | 0 | 0 | 0 | 0 | 0 | 0 | 0 | 0 |
| **Paronychia** | 0 | 0 | 0 | 1 | 0 | 0 | 0 | 0 | 0 | 0 | 0 | 0 |
| **Periodontitis** | 0 | 0 | 0 | 0 | 0 | 0 | 0 | 0 | 0 | 0 | 8 | 0 |
| **Pharyngitis** | 0 | 5 | 0 | 0 | 5 | 0 | 0 | 0 | 0 | 0 | 0 | 0 |
| **Pneumonia** | 0 | 5 | 0 | 1 | 17 | 0 | 0 | 0 | 0 | 0 | 2 | 2 |
| **Productive cough** | 0 | 1 | 0 | 0 | 0 | 0 | 0 | 0 | 0 | 0 | 0 | 0 |
| **Prostatic abscess** | 0 | 0 | 0 | 0 | 0 | 0 | 0 | 0 | 0 | 0 | 3 | 0 |
| **Pulmonary pain** | 0 | 0 | 0 | 0 | 5 | 0 | 0 | 0 | 0 | 0 | 0 | 0 |
| **Pyelonephritis** | 0 | 2 | 0 | 0 | 0 | 0 | 0 | 0 | 0 | 0 | 0 | 0 |
| **Pyrexia** | 0 | 4 | 0 | 0 | 8 | 0 | 0 | 1 | 0 | 0 | 0 | 0 |
| **Rash** | 0 | 15 | 0 | 0 | 7 | 0 | 0 | 1 | 0 | 0 | 0 | 0 |
| **Respiratory tract infection** | 0 | 20 | 0 | 1 | 3 | 0 | 0 | 1 | 0 | 0 | 5 | 0 |
| **Respiratory tract irritation** | 0 | 3 | 0 | 0 | 0 | 0 | 0 | 0 | 0 | 0 | 0 | 0 |
| **Rhinitis** | 0 | 7 | 0 | 0 | 16 | 0 | 0 | 0 | 0 | 0 | 0 | 0 |
| **Rhinorrhoea** | 4 | 24 | 0 | 0 | 3 | 0 | 0 | 0 | 0 | 0 | 2 | 0 |
| **Sinus pain** | 0 | 3 | 0 | 0 | 0 | 0 | 0 | 0 | 0 | 0 | 0 | 0 |
| **Sinusitis** | 0 | 3 | 0 | 0 | 19 | 0 | 0 | 0 | 0 | 0 | 8 | 0 |
| **Skin infection** | 16 | 4 | 0 | 0 | 4 | 0 | 0 | 0 | 0 | 0 | 5 | 0 |
| **Skin papilloma** | 0 | 0 | 0 | 0 | 3 | 0 | 0 | 0 | 0 | 0 | 0 | 0 |
| **Sneezing** | 0 | 0 | 0 | 0 | 0 | 0 | 0 | 0 | 0 | 0 | 2 | 0 |
| **Staphylococcal infection** | 0 | 0 | 0 | 0 | 1 | 0 | 0 | 0 | 0 | 0 | 0 | 0 |
| **Stomatitis** | 0 | 0 | 0 | 0 | 1 | 0 | 0 | 0 | 0 | 0 | 0 | 0 |
| **Swelling face** | 1 | 0 | 0 | 0 | 0 | 0 | 0 | 0 | 0 | 0 | 0 | 0 |
| **Swelling of eyelid** | 0 | 0 | 0 | 0 | 0 | 1 | 0 | 0 | 0 | 0 | 0 | 0 |
| **Throat irritation** | 0 | 6 | 0 | 0 | 0 | 0 | 0 | 0 | 0 | 0 | 0 | 0 |
| **Tinea faciei** | 0 | 2 | 0 | 0 | 0 | 0 | 0 | 0 | 0 | 0 | 0 | 0 |
| **Tongue fungal infection** | 0 | 0 | 0 | 0 | 0 | 0 | 0 | 0 | 0 | 0 | 2 | 0 |
| **Upper respiratory tract congestion** | 0 | 8 | 0 | 0 | 0 | 0 | 0 | 0 | 0 | 0 | 0 | 0 |
| **Upper respiratory tract infection** | 0 | 2 | 0 | 4 | 3 | 0 | 0 | 0 | 0 | 0 | 0 | 0 |
| **Upper respiratory tract inflammation** | 0 | 0 | 0 | 0 | 3 | 0 | 0 | 0 | 0 | 0 | 0 | 0 |
| **Urinary tract discomfort** | 0 | 9 | 0 | 0 | 0 | 0 | 0 | 0 | 0 | 0 | 0 | 0 |
| **Vomiting** | 0 | 1 | 0 | 0 | 0 | 0 | 0 | 0 | 0 | 0 | 0 | 0 |
| **Vulvitis** | 0 | 0 | 0 | 0 | 0 | 0 | 0 | 15 | 0 | 0 | 0 | 0 |
| **Vulvovaginal candidiasis** | 0 | 0 | 0 | 0 | 9 | 0 | 0 | 1 | 0 | 0 | 0 | 0 |
| **Wound infection** | 0 | 0 | 0 | 0 | 0 | 0 | 0 | 0 | 0 | 0 | 1 | 0 |
